# Changes in utilization and peri-operative outcomes of bariatric surgery in large U.S. hospital database, 2011-2014

**DOI:** 10.1371/journal.pone.0186306

**Published:** 2017-10-20

**Authors:** Lu Zhang, John Scott, Lu Shi, Khoa Truong, Qingwei Hu, Joseph A. Ewing, Liwei Chen

**Affiliations:** 1 Department of Public Health Sciences, Clemson University, Clemson, South Carolina, United States of America; 2 Department of Surgery, Greenville Health System, Greenville, South Carolina, United States of America; Stanford University School of Medicine, UNITED STATES

## Abstract

**Background:**

With the epidemic of morbid obesity, bariatric surgery has been accepted as one of the most effective treatments of obesity.

**Objective:**

To investigate recent changes in the utilization of bariatric surgery, patients and hospital characteristics, and in-hospital complications in a nationwide hospital database in the United States.

**Setting:**

This is a secondary data analysis of the Premier Perspective database.

**Methods:**

ICD-9 codes were used to identify bariatric surgeries performed between 2011 and 2014. Descriptive statistics were computed and regression was used.

**Results:**

A total of 74,774 bariatric procedures were identified from 436 hospitals between 2011 and 2014. During this time period, the proportion of gastric bypass (from 44.8% to 31.3%; P for trend < 0.0001) and gastric banding (from 22.8% to 5.2%; P for trend < 0.0001) decreased, while the proportion of sleeve gastrectomy (from 13.7% to 56.9%; P for trend < 0.0001) increased substantially. The proportion of bariatric surgery performed for outpatients decreased from 17.15% in 2011 to 8.11% in 2014 (P for trend < 0.0001). The majority of patients undergoing surgery were female (78.5%), white (65.6%), younger than 65 years (93.8%), and insured with managed care (53.6%). In-hospital mortality rate and length of hospital stay remained stable. The majority of surgeries were performed in high-volume (71.8%) and urban (91.6%) hospitals.

**Conclusions:**

Results based on our study sample indicated that the popularity of various bariatric surgery procedures changed significantly from 2011 to 2014. While the rates of in-hospital complications were stable, disparities in the use of bariatric surgery regarding gender, race, and insurance still exist.

## Introduction

The increasing prevalence of obesity, particularly morbid obesity, continues to be a serious public health concern in the United States (U.S.). The prevalence of morbid obesity increased 70% from 2001 to 2010 [1]. While diet and physical activity continue to be important efforts to curb the obesity epidemic in the current obesogenic environment, bariatric surgery has been an option for individuals with severe and morbid obesity and proven to be cost-effective [2]. In 1991, the National Institutes of Health (NIH) established guidelines for bariatric surgery for weight loss and recommended it to patients with body mass index (BMI) ≥ 40 or patients with BMI ≥ 35 with comorbidities [3]. Since then, the utilization of bariatric surgery has steadily increased in the U.S. [4–6].

Several studies investigated the trends and variations in the utilization of bariatric surgery [4, 6–9]. However, most published studies included the procedures performed in an inpatient setting and performed before 2012 [6, 7, 10, 11]. One most recent study looked at trends of bariatric surgery up to 2014 but did not investigate outpatient procedures, patients’ insurance coverage, and hospital characteristics [12]. Currently, outpatient procedures have been proven to be safe for several surgical types. For example, outpatient gastric banding has been widely accepted and sleeve gastrectomy has also been suggested to be safe when performed in an outpatient setting [13, 14]. Therefore, this study aimed to describe changes in the utilization of bariatric surgery in both inpatient and outpatient settings, by demographic characteristics of patients and types of hospitals, and in-hospital complications.

## Materials and methods

### Data source

Premier Perspective, one of the largest databases collecting standard hospital discharge files in the U.S., was used in our analysis. It includes both inpatient and outpatient visits from more than 600 hospitals [15]. The participating hospitals, from all regions of the U.S., could be teaching or nonteaching hospitals of varying sizes. Patients’ demographic information, diagnosis codes, medication, charges, hospital characteristics, and physician information were included in the Premier Perspective dataset.

### Patient selection

Patients aged 18 years or older who underwent bariatric surgery between January 1^st^, 2011 and June 30^th^, 2014 in the Premier Perspective dataset were included in the analysis. International Classification of Diseases, Ninth Revision, Clinical Modification (ICD-9) codes were used to identify bariatric procedures and were grouped into 4 categories including: 1) gastric bypass (44.31, 44.38, and 44.39), 2) sleeve gastrectomy (43.82), 3) gastric banding (44.95), and 4) other (43.7, 43.89, 44.68, 44.69, 45.50, 45.51, 45.90, and 45.91). To confirm the procedures as weight-loss surgery, only patients with obesity diagnosis (with one of following ICD-9 codes: 278.0, 278.00, 278.01, 278.03, 278.8, V77.8, and V85.30-V85.45) or with diagnosis-related group (DRG) code of 288 (indicating the primary reason for the hospital admission is weight-loss surgery) were included. Patients identified with abdominal neoplasm diagnosis (ICD-9 codes: 150.0–159.9) at hospital admission were excluded.

### Patient sociodemographics, hospital characteristics, and complications

Patients characteristics considered in this analysis included age, gender, race (non-Hispanic white, non-Hispanic black, and other), marital status (married, single, and other), and insurance (Medicare; Medicaid, charity or other government payors; managed care; commercial or employment related insurance; other). Comorbidities which identified by ICD-9 code at admission, included diabetes (250.xx), hypertension (401.0, 401.1, and 401.9), hyperlipidemia (272.0, 272.1, 272.2, and 272.4), chronic liver disease (070.22, 070.23, 070.32, 070.33, 070.44, 070.54, 070.6, 070.9, 456.0–456.2, 570.x, 571.x, 572.2–572.8, 573.3, 573.4, 573.8, 573.9, and V42.7), and sleep apnea (327.20–327.29, 780.51, 780.53, 780.57, and 786.03). Charlson Comorbidity Index (CCI) was calculated as Deyo’s enhanced ICD-9 coding algorithm [16]. Hospital procedure volume (number of bariatric surgeries per year) has been considered as one important criterion in bariatric surgery hospital accreditation, where 125 and 50 have been used as cutoff points [17, 18]. In our study, hospital volume was calculated as the total number of bariatric procedures performed in each hospital each year, then categorized into three groups: <50, 50–125, or >125 procedures per year. Because 2014 data was collected for only 6 months, the three categories of hospital volume in 2014 were adjusted to <25 procedures, 25–63 procedures, and >63 procedures, respectively. Other hospital characteristics included teaching status (teaching vs. non-teaching), hospital size (0–199, 200–499, or 500+ beds), and location (urban vs. rural area). Urban and rural areas were classified by U.S. Census Bureau [19, 20].

In-hospital mortality and length of hospital stay were calculated only among patients who received an inpatient procedure. For surgery complications, we employed the coding algorithms described in an article published in the Journal of the American Medical Association [6]. Surgery technical complications considered in the current study included hemorrhagic (998.11, 998.12, 99.04, 99.09) and wound complications (998.5, 998.51, 998.59, 998.13, 998.3). Surgery systemic complications included pulmonary (997.3, 481, 482.0–482.9, 485, 486, 518.81, 31.1, 31.29), cardiac (997.1, 410.0–410.9), neurological (997.01–997.03, 431.00–431.91, 433.00–433.91, 434.00–434.91, 436, 437.1), genitourinary tract (997.5, 584.1–584.9, 38.95, 39.95), thromboembolic complication (415.1, 415.11, 415.19, 453.8, 453.9), and postoperative shock (998.0) [6].

### Statistical analysis

Descriptive statistics including mean (± standard deviation) and percentage (%) were computed. Linear trend test was conducted by modeling the calendar year as a continuous predictor variable in linear regression or logistic regression, when appropriate. The significance test for coefficient of the calendar year was used as the statistical significance for trend analysis, reported as P for trend. All analysis was conducted using SAS 9.4 (SAS Institute, Inc., Cary, North Carolina).

## Results

### Types of procedures

From 4,197,408 hospital visits by patients with BMI ≥ 30 in the original dataset, a total of 74,774 bariatric procedures were identified. Gastric bypass was the most common surgery type in 2011 (44.76%) but decreased to the 2^nd^ most common procedure in 2014 (31.28%; P for trend < 0.0001) (Table 1). Meanwhile, gastric banding (100% performed laparoscopically) significantly decreased from 22.75% in 2011 to 5.22% in 2014 (P for trend < 0.0001), but the proportion of sleeve gastrectomy (100% performed laparoscopically) dramatically increased from 13.68% in 2011 to 56.85% (P for trend < 0.0001) and became the most commonly performed procedure in 2014.

**Table 1:** Bariatric surgery types and patient characteristics in Premier Prospective dataset, 2011–2014.

Characteristics	2011	2012	2013	2014 (6 months)	P for trend	Total
Total # of procedures	13,499	18,539	29,721	13,015		74,774
Surgery type, %						
Gastric bypass	44.76	43.59	35.50	31.28	<0.0001	38.45
Sleeve gastrectomy	13.68	35.03	50.37	56.85	<0.0001	41.07
Gastric banding	22.75	14.97	8.02	5.22	<0.0001	11.91
Other	18.81	6.40	6.11	6.65	<0.0001	8.57
Outpatient procedures, %	17.15	15.00	10.15	8.11	<0.0001	12.26
Age, mean±SD^†^, years	44.77±12.22	44.74±12.16	44.92±12.24	45.21±12.35	0.001	44.90±12.24
Age group, %						
18–39 years	35.73	36.27	35.35	34.91	0.04	35.57
40–54 years	41.00	40.64	40.89	40.91	0.96	40.85
55–64 years	17.89	17.30	17.44	17.15	0.18	17.43
≥65 years	5.39	5.79	6.33	7.04	<0.0001	6.15
Female, %	78.83	78.78	78.60	77.69	0.03	78.53
Race/Ethnicity, %						
Non-Hispanic white	63.10	67.25	65.83	65.19	0.02	65.57
Non-Hispanic black	15.25	14.99	14.49	13.79	0.0003	14.63
Other	21.65	17.76	19.69	21.02	0.73	19.80
Married, %	46.58	49.33	48.35	46.15	0.30	47.89
Insurance, %						
Medicare	15.82	15.38	17.17	19.20	<0.0001	16.84
Medicaid or other government payors	11.49	11.76	12.49	13.02	<0.0001	12.22
Managed care	56.14	54.68	53.12	50.31	<0.0001	53.61
Commercial or employment related	11.37	12.13	12.00	12.10	0.11	11.94
Other	5.19	6.05	5.11	5.37	0.27	5.40
Comorbidity before surgery, %						
Diabetes	31.50	31.12	31.25	31.14	0.63	31.24
Hypertension	54.47	54.12	54.09	55.04	0.48	54.33
Hyperlipidemia	37.91	37.34	38.14	38.43	0.15	37.95
Chronic liver disease	9.80	9.87	10.68	10.04	0.06	10.21
Sleep apnea	42.92	43.25	43.24	44.31	0.05	43.37
Charlson Comorbidity Index, %						
0	45.92	45.58	43.64	43.23	<0.0001	44.46
1	32.72	31.80	31.48	32.44	0.35	31.95
≥2	21.36	22.62	24.88	24.33	<0.0001	23.59

^†^Standard deviation.

**Fig 1:**
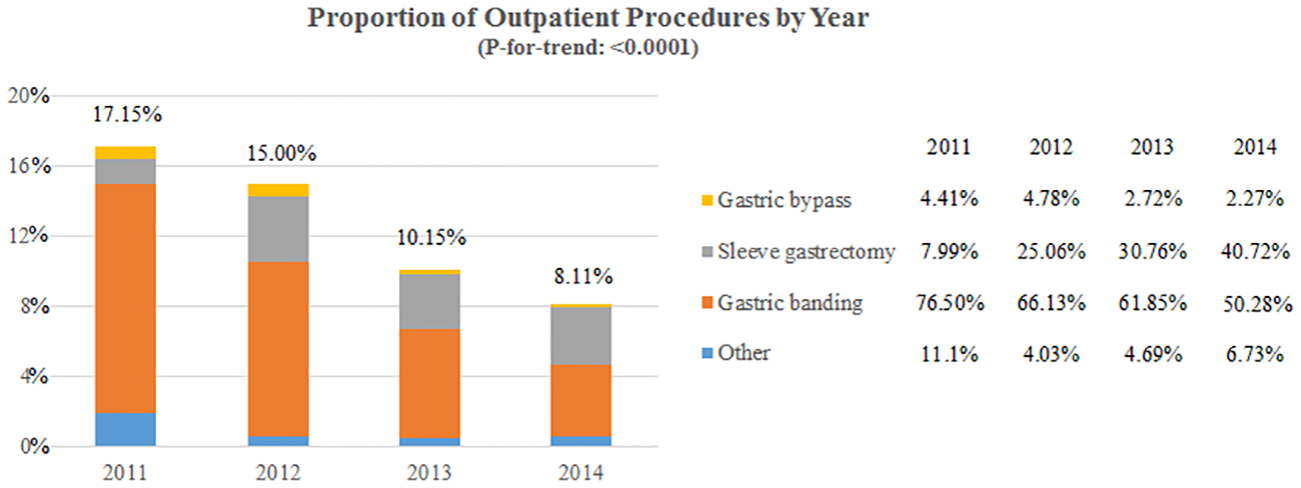
Proportion of outpatient procedures from 2011 to 2014.

The proportion of bariatric surgery procedures performed in outpatient settings decreased from 17.15% in 2011 to 8.11% in 2014 (P for trend < 0.0001). Among all outpatient procedures, the proportion of each surgery type varied across years (Fig 1). For example, the proportion of sleeve gastrectomy increased from 7.99% to 40.72%; but gastric banding decreased from 76.50% to 50.28% between 2011 and 2014.

### Patient characteristics

The mean age of patients undergoing bariatric surgery increased slightly, from 44.77 years (SD = 12.22) in 2011 to 45.21 years (SD = 12.35) in 2014 (P for trend = 0.001) (Table 1). The percentage of older patients (≥65 years) increased from 5.39% in 2011 to 7.04% in 2014 (P for trend < 0.0001). Overall, the majority of patients were female (78.53%) and non-Hispanic white (65.57%). The percentage of female patients decreased slightly over time (from 78.83% to 77.69%; P for trend = 0.03) but the percentage of white patients increased (from 63.10% to 65.19%; P for trend = 0.02) from 2011 to 2014. Almost half of patients were married (47.89%) and the percentage did not change over time (P for trend = 0.30). More than half of patients (53.61%) were covered by managed care, but the proportion decreased from 56.14% to 50.31% (P for trend < 0.0001). The proportion of procedures covered by Medicare and Medicaid increased from 15.82% to 19.20% (P for trend < 0.0001) and from 11.49% to 13.02% (P for trend < 0.0001), respectively. Overall, 31.24% patients had diabetes, 54.33% had hypertension, 37.95% had hyperlipidemia, 10.21% had chronic liver disease, and 43.37% had sleep apnea. The prevalence of the comorbidities among surgery patients remained stable over time except for sleep apnea (42.92% in 2011 vs. 44.31% in 2014; P for trend = 0.05) (Table 1). The proportion of patients with a CCI score of 0 decreased from 45.92% in 2011 to 43.23% in 2014 (P for trend < 0.0001), while the proportion of patients with a CCI score ≥2 increased from 21.36% to 24.33% (P for trend < 0.0001).

### Hospital characteristics

A total of 436 hospitals performing bariatric surgery between January 1^st^ 2011 and June 30^th^ 2014 were included in this analysis. The distribution of hospitals by volumes of bariatric procedures changed over time: low-volume hospitals (<50 procedures per year) decreased from 66.90% in 2011 to 54.91% in 2014 and high-volume hospitals (>125 procedures per year) increased from 13.88% to 29.09% (data were not shown in the tables). The other hospital characteristics remained consistent over time: teaching hospitals were 31.65%, hospitals with 500+ beds were 16.51%, and urban hospitals were 80.50%.

About half of the procedures were performed in teaching hospitals (decreasing from 52.14% in 2011 to 45.13% in 2014; P for trend < 0.0001) and a majority of the procedures were performed in urban hospitals (decreasing from 92.94% in 2011 to 90.68% in 2014; P for trend < 0.0001) (Table 2). In 2011, 53.55% of bariatric surgeries were performed in high-volume hospitals and 14.22% were performed in low-volume hospitals. The gap became wider in 2014, with 79.89% of bariatric surgeries performed in high-volume hospitals and only 6.41% performed in low-volume hospitals. As for hospital bed size, about half (48.28%) of the surgeries were performed in middle-sized hospitals (200–499 beds). The proportion of procedures performed at large hospitals (500+ beds) was consistent over time (33.10% in 2011 and 31.79% in 2014; P for trend = 0.45). Meanwhile the proportion of procedures performed in small hospitals (<200 beds) increased from 16.65% in 2011 to 21.52% in 2014 (P for trend < 0.0001) and the proportion of procedures performed in middle-sized hospitals decreased from 50.26% in 2011 to 46.68% in 2014 (P for trend < 0.0001).

**Table 2:** Bariatric surgery utilization by hospital characteristics in Premier Prospective dataset, 2011–2014.

Hospital Characteristics	2011	2012	2013	2014	P for trend	Total
Hospital volume, %						
<50 procedures per year	14.22	9.16	5.55	6.41	<0.0001	8.16
50–125 procedures per year	32.23	22.59	15.81	13.70	<0.0001	20.09
>125 procedures per year	53.55	68.25	78.64	79.89	<0.0001	71.75
Teaching hospital, %	52.14	51.29	47.52	45.13	<0.0001	48.87
Bed size, %						
0–199	16.65	18.33	19.59	21.52	<0.0001	19.08
200–499	50.26	49.77	47.15	46.68	<0.0001	48.28
≥500	33.10	31.89	33.27	31.79	0.45	32.64
Urban area, %	92.94	92.84	90.53	90.68	<0.0001	91.56

### In-hospital complication

As shown in Table 3, common surgery complications remained stable over time. Overall, the in-hospital mortality was 0.29% and the average length of hospital stay was 2.55 days (SD = 5.39). The percentage of systemic complications was 2.94% (pulmonary: 1.43%; cardiac: 0.40%; neurological: 0.04%; urinary tract: 1.51%; thromboembolic: 0.16%; post-operative shock: 0.10%) and did not change significantly over the four-year period. The percentage of hemorrhagic complication decreased from 2.66% in 2011 to 2.36% in 2014 (P for trend = 0.03) but no significant difference was observed for wound complication (P for trend = 0.94).

**Table 3:** Outcomes of bariatric surgery in Premier Prospective dataset, 2011–2014.

Surgery Outcome	2011	2012	2013	2014	P for trend	Total
In-hospital mortality^†^, %	0.35	0.34	0.25	0.28	0.13	0.29
Length of hospital stay^†^, mean±SD^‡^, days	2.57±5.86	2.61±5.24	2.54±5.39	2.49±5.11	0.14	2.55±5.39
Technical complication, %						
Hemorrhagic complication	2.66	2.70	2.43	2.36	0.03	2.53
Wound complication	0.48	0.60	0.49	0.53	0.94	0.52
Systemic complication, %						
Pulmonary complication	1.47	1.50	1.43	1.26	0.12	1.43
Cardiac complication	0.30	0.47	0.42	0.35	0.65	0.40
Neurological complication	0.04	0.04	0.05	0.05	0.44	0.04
Urinary tract complication	1.43	1.60	1.52	1.43	0.87	1.51
Thromboembolic complication	0.19	0.12	0.16	0.18	0.86	0.16
Post-operative shock	0.07	0.12	0.10	0.12	0.32	0.10

^†^In-hospital mortality and length of hospital stay were calculated only among patients receiving inpatient procedures.

^‡^Standard deviation.

## Discussion

In this study, we described the changes in the utilization of bariatric surgery using a large hospital database from 2011 to 2014. We observed a continuous decline in the use of gastric bypass and gastric banding, but a dramatic increase in sleeve gastrectomy, which accounted for more than half of bariatric procedures in 2014. The utilization of outpatient procedures decreased and the proportion of each surgery type in outpatient settings varied during the study period. A majority of the surgeries were performed in urban hospitals. The proportion of procedures covered by Medicare or Medicaid and the procedures performed in high-volume hospitals increased consistently over the four-year period. Minor changes were observed for patients’ comorbidity and in-hospital complications.

Several studies evaluated the change in bariatric surgery types in the U.S. over time [7, 9, 10, 12, 21, 22]. Gastric banding was a popular procedure in the early 2000’s (accounting for 25–30% of all bariatric surgeries) [9] because of its safety and reversibility, which allows for the removal of original banding and the access to other surgical options [21]. However, it has also been shown that up to 20% of gastric banding procedures may require some form of revisional procedures during the first 10 years [21] and its popularity has decreased since 2008 [7, 9]. Sleeve gastrectomy, which showed favorable peri-operative outcomes, superior early effectiveness in weight loss, and less concerns for long-term risks, has become more popular in recent years [21]. Previous studies identified a reduction in the use of gastric banding parallel with a remarkable increase in the use of sleeve gastrectomy from 2008 to 2012 [7, 9, 10]. It was reported that 5.6% and 39.6% of all bariatric procedures were gastric banding and sleeve gastrectomy in the period of 2008–2012, respectively [10]. One recent study using data from 2010 to 2014 reported the continuous increase in the proportion of sleeve gastrectomy to 58.2% in 2014 [12]. Our results were consistent with their findings showing the proportion of sleeve gastrectomy increasing from 35.0% in 2012 to 56.9% in 2014.

To our knowledge, our study is the first to investigate the proportion of outpatient bariatric surgery. We found that the proportion of all outpatient surgeries decreased from 17.15% to 8.11% between 2011 and 2014. A potential explanation for this could be related to the predominant surgery type in this period. In an outpatient setting, gastric banding was the earliest and most widely accepted surgery type [23], while sleeve gastrectomy has been suggested to be safe as a new surgery type [24]. About 78.20% of gastric banding surgeries and 5.81% of sleeve gastrectomy surgeries were performed as outpatient procedures in 2014 in our study (data were not shown in Fig 1). However, the proportion of all outpatient procedures decreased from 76.50% to 50.28% for gastric banding and increased from 7.99% to 40.27% for sleeve gastrectomy between 2011 and 2014, which reflects the transition of the popularity of bariatric surgery types.

Consistent with the literature, the majority of bariatric surgery patients in our study were female, younger, and non-Hispanic white. We observed diminishing differences in the age and gender but slightly increasing difference in race over the study period. Previous research reported that compared to whites, blacks were more likely to be eligible for bariatric surgery but less likely to receive it [25]. Underlying reasons of these racial differences are complex. Unequal access to health care could partially explain the disparity where lower insurance coverage rate among black patients than among white patients was reported [25] and lack of insurance coverage was the most frequent reason of rejecting bariatric surgery [26]. Another possible explanation, from the social norm perspective, is that black people have a more positive attitude towards larger body size [27], therefore they may be less willing to take the surgery risk. Although bariatric surgery has been proven safe, concerns about surgical complications or death remain the most common reason for not considering bariatric surgery [28].

We found that the proportion of bariatric surgeries performed in high-volume hospitals (over 125 procedures per year) increased over time. Procedure volume was a major criterion in the hospital accreditation, where a minimum of 125 procedures per year was required to be classified as a Centers of Excellence (COE) for bariatric surgery [17]. In February 2006, the Centers for Medicare and Medicaid Services (CMS) issued a National Coverage Determination (NCD), which restricted insurance coverage for bariatric surgeries only to COE hospitals [29]. In September 2013, however, CMS changed the policy and removed the restriction of coverage to COE hospitals [29]. In the following year, Metabolic and Bariatric Surgery Accreditation and Quality Improvement Program (MBSAQIP), which is jointly supported by American College of Surgeons (ACS) and American Society for Metabolic and Bariatric Surgery (ASMBS), changed the evaluation criteria on hospital volume from 125 to 50 procedures [18]. One study analyzing in-patient claims from two states found that the percentage of procedures performed at COE hospitals increased from 60.5% in 2008 to 73.1% in 2011 [30]. In our study, the percentage of procedures performed in high-volume hospitals increased from 53.6% in 2011 to 79.9% in 2014. The observed increase of frequency of bariatric procedures in high-volume hospitals might be the result of restriction of insurance coverage to COE hospitals.

One significant concern regarding NCD is whether it impaired the access to bariatric surgery for some underserved populations. Despite lacking information on hospitals’ COE status, we found that the proportion of bariatric procedures covered by Medicare or Medicaid increased modestly and steadily in our study sample between 2011 and 2014. Although several studies reporting that patients without private insurance were significantly underrepresented in bariatric surgery [31, 32], our finding of the improved access to bariatric surgery for Medicare or Medicaid beneficiaries is encouraging. In a national representative dataset, the percentage of bariatric procedures covered by Medicare rose from 8.5% in 2006 to 16.3% in 2011 [33], while increasing from 15.8% in 2011 to 19.2% in 2014 in our study. One recent study also reported that patients covered by Medicare or Medicaid are more likely to undergo gastric bypass than privately insured patients [34]. Although it is not our intention to evaluate the impact of NCD, the findings from previous studies along with our study collectively suggest that access to bariatric surgery, particularly for Medicare or Medicaid beneficiaries, did not decrease over the past few years.

The percentage of most selected comorbidities (diabetes, hypertension, hyperlipidemia, and chronic liver disease) was stable from 2011 to 2014 in our study. Sleep apnea was an exception with a slight increase. Bariatric surgery has been proven as an effective treatment for type 2 diabetes [35]. Previous research has shown the proportion of bariatric surgery patients who had type 2 diabetes increased greatly from 1998 (14%) until 2008 (33%) but plateaued after that. In our data, about 31.2% patients had type 2 diabetes over the study period, which is similar to previous findings [7, 10]. One previous study reported an upward trend of the sleep apnea among bariatric surgery patients from 28% in 2008 to 35.8% in 2012 [10]. The proportion of sleep apnea in our data was higher than previously reported and increased continuously [7, 10].

The median length of hospital stay was 2 days in our study, which is consistent with other reports [7, 10]. Except the decreasing frequency of hemorrhagic complication, the frequency of other technical or systemic complications remained stable over time. Compared to a study evaluating the same type of in-hospital complications from 1998 to 2002 [6], our study identified substantially lower rates of wound, pulmonary, and cardiac complications between 2011 and 2014, suggesting improvements have been made. This observation agrees with recent studies [10]. However, in-hospital mortality rate stayed relatively high, from 0.35% to 0.28% over the four years, compared to 0.1% during 2008 and 2012 in other studies [7, 10]. Since we could not identify any other estimated mortality of bariatric surgery mortality during the same period (2011–2014), it is not possible to conclude whether the increase in mortality rate reflects a temporal change or is strictly related to patients and bariatric surgery of our study sample. Future studies may be needed to shed further light on mortality of bariatric surgery.

Premier Prospective dataset is one of the largest hospital discharge datasets in the US. The primary strength of this dataset is the inclusion of both inpatient and outpatient visits. Compared to most descriptive studies using National Inpatient Sample (NIS) dataset, which limits to inpatient bariatric surgery [6, 7, 10, 11], our study was able to capture the outpatient procedures. Despite its strengths, Premier Prospective dataset has major limitations. First, it is not nationally representative (no sampling strategy was employed in data collection). Although the proportions of female patients (79% vs 78%), white patients (63% vs. 62%), and Medicare beneficiaries (19% vs. 18%) in 2011 in our study were similar to those previously reported using NIS dataset [7, 30, 33], interpretation of the findings from this study needs caution. Second, the database does not have information to identify patients who transferred from outpatient to inpatient facilities. The prevalence of outpatient procedures therefore could be slightly underestimated, although the proportion of hospitalization for outpatient procedures is very low—less than 2% [14]. Third, to select the bariatric procedures as weight-loss surgery, we included patients with morbid obesity diagnosis or with DRG code of 288. DRG code of 288 indicates the primary reason for the hospital admission as weight-loss surgery, however, in preliminary analysis we found that half of patients who had bariatric surgery and morbid obesity diagnosis were not coded with DRG of 288. To preserve sample size and in the concern of underuse of DRG code, we did not restrict our sample to only the patients who had DRG code of 288. Fourth, since the ICD-9 code for sleeve gastrectomy became available in October 2011, the proportion of sleeve gastrectomy in 2011 could be underestimated in this study. Our results were very close to those estimated from the nationally representative data [7] though (14% vs. 12%). In addition, if we only compare the proportion of sleeve gastrectomy in 2012 (35%) to that in 2014 (57%), the increase was still substantial. Lastly, only in-hospital mortality and peri-operative complications were identified. Post-discharge complications or mortality were not captured in this dataset.

## Conclusions

In this study, we observed the change in the bariatric surgery types as well as the changes in patient and hospital characteristics in recent years provided in a large U.S. hospital database, Premier Perspective. Except for a slight decrease in the proportion of hemorrhagic complication, we did not find changes in hospital mortality and other peri-operational complications. Most procedures are performed in high-volume hospitals. Sex and racial disparities in bariatric surgery appear consistent. Findings from this study points to the direction that effort should be made to increase access to bariatric surgery with regarding to gender, race, and insurance type.
